# Influence of Nutrition, Food and Diet-Related Interventions in the Workplace: A Meta-Analysis with Meta-Regression

**DOI:** 10.3390/nu13113945

**Published:** 2021-11-04

**Authors:** Liliana Melián-Fleitas, Álvaro Franco-Pérez, Pablo Caballero, María Sanz-Lorente, Carmina Wanden-Berghe, Javier Sanz-Valero

**Affiliations:** 1 Nutrition Department, University of Granada, 18012 Granada, Spain; lilianamelian@hotmail.es; 2 Geriatric Service, Insular Hospital, Health Services Management of the Health Area of Lanzarote, 35500 Arrecife, Spain; 3 Playa Blanca Health Center, Health Services Management of the Health Area of Lanzarote, 35580 Playa Blanca, Spain; amoisesfp@hotmail.com; 4 Department of Community Nursing, Preventive Medicine and Public Health and History of Science, University of Alicante, San Vicente del Raspeig, 03690 Alicante, Spain; pablo.caballero@ua.es; 5 Department of Public Health & History of Science, University Miguel Hernandez, 03550 Alicante, Spain; msanzlor@gmail.com; 6 Center of Public Health, Consellería of Universal Health and Public Health, 46940 Manises, Spain; 7 Health and Biomedical Research Institute of Alicante, University General Hospital, 03010 Alicante, Spain; carminaw@telefonica.net; 8 National School of Occupational Medicine, Carlos III Health Institute, 28029 Madrid, Spain

**Keywords:** diet, food, nutrition, occupational health, working conditions, workplace, obesity, overweight, occupational health policy

## Abstract

Objective: To review the scientific literature on the influence of verified nutrition, food and diet interventions on occupational health. Method: This study involved a critical analysis of articles retrieved from MEDLINE (via PubMed), Embase, Cochrane Library, PsycINFO, Scopus, Web of Science, Latin American and Caribbean Health Sciences Literature (LILACS) and Medicina en Español (MEDES) using the descriptors “Diet, Food, and Nutrition” and “Occupational Health” and applying the filters “Clinical Trial”, “Humans” and “Adult: 19+ years”; the search was conducted on 29 May 2021. Results: A total of 401 references were retrieved from the bibliographic databases, with an additional 16 identified through a secondary search; among the studies retrieved, 34 clinical trials were selected after applying the inclusion and exclusion criteria. The interventions were grouped into seven categories: (1) dietary interventions associated with exercise or educational programs; (2) individual environmental interventions or other educational actions; (3) educational interventions oriented toward lifestyle, dietetics, physical activity and stress management; (4) economic incentives; (5) multicomponent interventions (combination of mindfulness, e-coaching and the addition of fruits and vegetables); or dietary interventions (facilitating greater food supply in cafeterias); or interventions focused on physical exercise. Conclusions: Given that most people spend a large part of their time in the workplace and, therefore, eat at least one of their daily meals there, well-planned interventions—preferably including several strategies—have been demonstrated, in general, as useful for combating overweight and obesity. From the meta-regression study, it was observed that the interventions give better results in people who presented high Body Mass Index (BMI) values (obesity). In contrast, intervention 2 (interventions related to workplace environment) would not give the expected results (it would increase the BMI).

## 1. Introduction

The importance of good health, physical activity and adequate nutrition is frequently discussed. However, there are many occasions in which we do not realize that health and work go hand-in-hand, influencing each other. In this relationship, it must be taken into account that a large number of people eat at least one of their daily meals in the workplace, which makes food very important in working life.

A proper diet together with adequate hydration has the potential to influence many aspects of work. However, well-designed nutritional interventions as measures to improve the health and performance of workers are scarce [1]. Importantly, nutrition is an essential part of economic development because it influences the health and productivity of workers [2,3].

Community health focuses on the influence of adequate food/nutrition (diet) on occupational health and how to address dietary limitations (malnutrition) and dietary excesses (obesity). In this sense, the National Institute for Occupational Safety and Health (NIOSH), which is part of the US Centers for Disease Control and Prevention, aims to raise awareness among employers and empower workers to create safe and healthy workplaces. NIOSH encourages “Total Worker Health”, a strategy that integrates occupational safety and health protection to prevent worker injuries and illnesses and improve their health and well-being, with access to healthy and affordable food being an important topic [4].

The worldwide prevalence of overweight and obesity has tripled since the mid-1970s. Data for 2016 show that more than 1.9 billion adults were overweight, of whom more than 650 million were obese [5]. This trend is based on overeating, sedentary behaviors, unhealthy lifestyles, insufficient levels of physical activity, poor diet (highly caloric and processed foods), as well as a higher proportion of sedentary occupations [6,7].

Obesity significantly increases the risk of developing metabolic disorders, hypertension, coronary heart disease, stroke, dyslipidemia, type 2 diabetes, sleep apnea, acute respiratory distress syndrome and several types of cancer. In addition, it is associated with an increase in mortality and a low quality of life [8].

Additionally, this morbidity is also related to indirect costs, defined as losses due to reduced labor productivity. In fact, obesity and related diseases have been associated with an increased risk of workplace absenteeism (refers to the time taken off work due to sick leave, disability, injuries, or other reasons), presenteeism (refers to situations where people continue to work while unwell and not functioning to their full capacity) and permanent loss of work, which includes pensions for disability and premature death, generating massive costs for governments, society and employers [7,9]. In fact, productivity losses due to sick leave and presenteeism are even greater than the direct costs of medical treatment (an average of 2.30 USD in lost productivity for every dollar in medical expenses) [10].

This evidence urges governments, scientific organizations and companies to implement occupational safety and health measures, policies and global strategies that focus on organizational, behavioral and environmental factors related to work and that directly influence the overall health of workers and companies, paying special attention to nutrition. Consequently, companies and institutions have the responsibility of ensuring that the foods available in the workplace are nutritionally adequate or making unhealthy options unavailable.

Among the current trends focused on nutrition and occupational health, the creation of a new concept, nutra-ergonomics, stands out. Nutra-ergonomics is defined as the interface between workers, their work environment and their performance in relation to their nutritional status. Nutrition is an integral part of a safe and productive workplace that encompasses physical and mental health as well as the long-term well-being of workers [1].

From this global perspective, health and well-being programs in the workplace are presented as the best tools to address this growing problem; these programs comprise a set of coordinated strategies (including programs, policies, benefits, environmental support and links to the surrounding community) that are implemented in the workplace, designed to improve the health and safety of all employees [11], and there are studies that support the effectiveness of these programs in improving employee health and productivity [1,11,12].

In addition, there is a general consensus that the combination of multicomponent interventions (focused on lifestyle management that includes stress management, physical activity, nutrition, and controlling smoking and alcohol consumption) is more effective than programs that focus on a single intervention (only exercise, for example) [13,14].

The objective of this review was to review the scientific literature on the influence of verified nutrition, food and diet interventions on occupational health.

## 2. Materials and Methods

### 2.1. Design

This was a cross-sectional descriptive study and critical analysis of studies retrieved through a systematic review. The structure of this review followed the Preferred Reporting Items for Systematic Reviews and Meta-Analyses (PRISMA) guidelines, and the methodological framework proposed by Arksey & O’Malley [15] for scoping studies.

### 2.2. Source of Data Collection

The data were obtained from direct consultation and access, via the internet, to the following bibliographic databases in the field of health sciences: MEDLINE (via PubMed), Embase, Cochrane Library, PsycINFO, Scopus, Web of Science, and Latin American & Caribbean Health Sciences Literature (LILACS) and Medicina en Español (MEDES).

### 2.3. Unit of Analysis

We analyzed articles published and retrieved from the indicated bibliographic databases.

### 2.4. Information Processing

Search terms were selected using the Thesaurus of Health Sciences Descriptors (DeCS) developed by the Latin American and Caribbean Center on Health Sciences Information (BIREME) and equivalent terms established by the US National Library of Medicine, Medical Subject Headings (MeSH).

Based on the hierarchy of both thesaurus and their indexing files, the following search equations were considered adequate:

- Equation (1): Occupational Health

“Occupational Health” [Mesh] OR “Occupational Health” [Title/Abstract] OR “Industrial Hygiene” [Title/Abstract] OR “Industrial Health” [Title/Abstract] OR “Occupational Safety” [Title/Abstract] OR “Employee Health” [Title/Abstract] OR “Occupational exposure” [Mesh] OR “Occupational exposure” [Title/Abstract] OR “Occupational stress” [Mesh] OR “Occupational stress” [Title/Abstract] OR “Occupational diseases” [Mesh] OR “Occupational diseases” [Title/Abstract] OR “Occupational hazards” [Title/Abstract] OR “Occupational medicine” [Mesh] OR “Occupational medicine” [Title/Abstract] OR “Occupational health safety” [Title/Abstract] OR “Occupational Health Services” [Title/Abstract] OR “Occupational Health Services” [Mesh] OR “National Institute for Occupational Safety and Health (U.S.)” [Mesh] OR “Occupational stressors” [Title/Abstract] OR “Occupational stressor” [Title/Abstract] OR “Occupational Factors “[Title/Abstract] OR “Workplace” [Mesh] OR “Workplace” [Title/Abstract] OR “Workplace Health” [Title/Abstract] OR “Workplace safety” [Title/Abstract] OR “Safety climate” [Title/Abstract] OR “Total worker health” [Title/Abstract] OR “Working Environment” [Title/Abstract] OR “Job Satisfaction” [Mesh] OR “Job Satisfaction” [Title/Abstract] OR “Job Stress” [Title/Abstract] OR “Job security” [Title/Abstract] OR “Psychosocial working conditions” [Title/Abstract] OR “Employee Health” [Title/Abstract].

- Equation (2): Diet, Food, and Nutrition

“Diet, Food, and Nutrition” [Mesh] OR “Nutritional Status” [Mesh] OR “Nutritional Status” [Title/Abstract] OR “Nutrition Therapy” [Mesh] OR “Nutrition Therapy” [Title/Abstract] OR “Nutrition Assessment” [Mesh] OR “Nutrition Assessment” [Title/Abstract] OR “Nutrition Surveys” [Mesh] OR “Nutrition Surveys” [Title/Abstract] OR “Diet” [Mesh] OR “Diet” [Title/Abstract] OR “Healthy Diet” [Mesh] OR “Healthy Diet” [Title/Abstract] OR “Healthy Eating” [Title/Abstract] OR “Energy Intake” [Mesh] OR “Energy Intake” [Title/Abstract] OR “Meals” [Mesh] OR “Meals” [Title/Abstract] OR “Meal Time” [Title/Abstract] OR “Dinner Time” [Title/Abstract] OR “Breakfast” [Mesh] OR “Breakfast” [Title/Abstract] OR “Breakfast Time “[Title/Abstract] OR “Morning Meal” [Title/Abstract] OR “Food Services” [Mesh] OR “Food Services” [Title/Abstract] OR “Eating Practices” [Title/Abstract] OR “Dietary practices” [Title/Abstract] OR “Unhealthy food options” [Title/Abstract] OR “Eat and drink” [Title/Abstract] OR “Meal breaks” [Title/Abstract] OR “Dietary habits” [Title/Abstract] OR “Eating behavior” [Title/Abstract] OR “Meal timing” [Title/Abstract] OR “Eating at night” [Title/Abstract] OR “Body weight” [Title/Abstract] OR “BMI” [Title/Abstract] OR “Shiftwork” [Title/Abstract] OR “Work Hygiene” [Title/Abstract] OR “Healthy Lifestyle” [Mesh] OR “Feeding Behavior” [Mesh] OR “Feeding Behavior” [Title/Abstract] OR “Feeding Behaviors” [Title/Abstract] OR “Eating Behaviors” [Title/Abstract] OR “Feeding Patterns” [Title/Abstract] OR “Feeding Pattern” [Title/Abstract] OR “Food Habits” [Title/Abstract] OR “Food Habit” [Title/Abstract] OR “Eating Habits” [Title/Abstract] OR “Eating Habit” [Title/Abstract] OR “Diet Habits” [Title/Abstract] OR “Diet Habit” [Title/Abstract].

The final search equation was developed for use in MEDLINE via PubMed through the Boolean union of the 2 proposed equations (Equation (1) AND Equation (2)) using the filters Clinical Trial, Humans and Adult: 19+ years.

This strategy was subsequently adapted to the characteristics of each of the other databases consulted, performing the search from the first available date in each of the selected databases until 29 May 2021. Additionally, a complementary search strategy was performed to reduce the possibility of publication bias by manually searching the reference lists of the clinical trials that were selected for the review. Likewise, experts in the subject under study were contacted to determine the possible existence of gray literature (materials and research produced by organizations outside traditional commercial or academic publications that are disseminated through other distribution channels).

### 2.5. Final Selection of Articles

For the review and critical analysis, articles that met the following criteria were chosen:

- Inclusion: met the objectives of the search; clinical trial; published in a peer-reviewed journal and written in English, Spanish or Portuguese.
- Exclusion: full text could not be found; no relationship between the intervention and the outcome under study (causality criterion), and included a nonadult population (under 18 years of age).

The selection of relevant articles was performed by two authors of the present review (L.M-F. and A.F-P.). To validate the inclusion of the articles, the assessment of the agreement between the authors (kappa index = KI) had to be greater than 0.60 [16]. Provided that this condition was met, possible disagreements were resolved by consensus among all authors of the review.

### 2.6. Completeness of Reporting, Level of Evidence and Grade of Recommendation

The adequacy of the selected articles was assessed using the CONSORT (Consolidated Standards of Reporting Trials) guidelines for reporting clinical trials [17]. This checklist contains 25 essential elements (items) that should be described in this type of study. One point was assigned for each item present (if not applicable, the item was not scored). When an item comprised several points, each was evaluated independently, giving the same weight to each point, and then the points for the item were averaged to obtain a final result, therefore, in no case was it possible to score more than 1 point per item.

To determine the level of evidence and its degree of recommendation, the recommendations of the Scottish Intercollegiate Guidelines Network Grading Review Group (SIGN) [18] were used.

### 2.7. Data Extraction

Data correction was performed by inputting the data into duplicate tables, thus allowing the detection of deviations and their correction through consultation of the original document.

The elimination of duplicate records (present in more than one database) was executed using the multiplatform program ZOTERO (bibliographic reference manager developed by the Center for History and New Media of George Mason University).

To determine the actuality of the studies, the Burton–Kebler half-period (BK) and the Price index (PI) were calculated.

The articles were grouped based on the variables under study to systematize and facilitate the understanding of the results, considering the following data: first author, year of publication, population studied, pathology of the population, country where the study was developed, period of the study, type of intervention performed, and main results influenced by the effect of the intervention.

### 2.8. Data Analysis

Data related to information retrieval are presented as frequencies and percentages.

To determine the BK, the median age was calculated based on the time range analyzed, and the PI was calculated as the percentage of articles 5 years old or newer.

The measure of agreement to determine the relevance of the selected articles was performed using the KI. The agreement between authors was considered valid when the KI value was greater than 60% (good or very good agreement).

The scores of the CONSORT questionnaire were analyzed using the median, maximum and minimum. The evolution of this score, in relation to the years of publication, was obtained by Pearson’s correlation analysis.

### 2.9. Meta-Analysis and Meta-Regression

To find out the effects of the interventions on workers’ BMI, we analyzed the effect size using a meta-analysis of the studies included in the systematic review. The estimated model was the random-effects model. The results of the effect size and its 95% confidence interval were presented in the Forest plot, together with the percentage of heterogeneity, the Tau value for the contrast and the corresponding heterogeneity test.

Publication bias occurs when only favorable results are published, and it is suspected that studies with non-significant results failed to be published. The absence of such studies may overestimate the results. In this study, the Funnel plot has been used. In the Funnel plot, the effect measure of each study is plotted on the *x*-axis and a measure of precision, such as the standard error on the *y*-axis. A meta-analysis without publication bias would show a point cloud in the shape of an inverted funnel. Based on this assumption, we performed the non-parametric trim-and-fill analysis proposed by Duval and Tweedie [19], adjusting for the number of missing studies and re-estimating the results by including these missing studies. Another approach to estimating the number of missing studies was proposed by Copas et al. [20], which we have also used.

Meta-regression was used to determine whether intervention type or baseline BMI status would influence heterogeneity and effect sizes. Bivariate and multivariate models were applied. Baseline BMI status was divided into three groups, normal weight, overweight and obese, and five interventions were studied.

The results of the articles selected from the systematic review are shown by their authors in three different ways: the results before and after the interventions in terms of mean and standard deviation, the difference between before and after the interventions in terms of mean and 95% CI and finally, the difference between before and after the interventions in terms of mean and standard deviation. In order to unify the criteria, the last option was used in the meta-analysis. Therefore, for the first situation, the difference of means and the weighted standard deviation were calculated for the first case. For the second case, the estimated standard error was obtained from the width of the 95% CI and the sample size.

All calculations were performed in the R programming environment using the packages meta version 4.10-0 and metas version 0.4-0 [21].

### 2.10. Ethical Aspects

All data were obtained from published articles. Therefore, and in accordance with Spanish Law 14/2007, approval by an ethics committee was not necessary for the use of secondary data.

## 3. Results

A total of 401 articles were retrieved: 121 (30.17%) in MEDLINE (via PubMed), 47 (11.72%) in Embase, 62 (15.46%) in Cochrane Library, 82 (20.45%) in Scopus, 33 (8.23%) in Web of Science, 50 (12.47%) in PsycINFO and six (1.502%) in MEDES. No documents were found in the LILACS bibliographic database. Consultation of the bibliographic lists of selected articles allowed the identification of another 16 studies.

**Figure 1:**
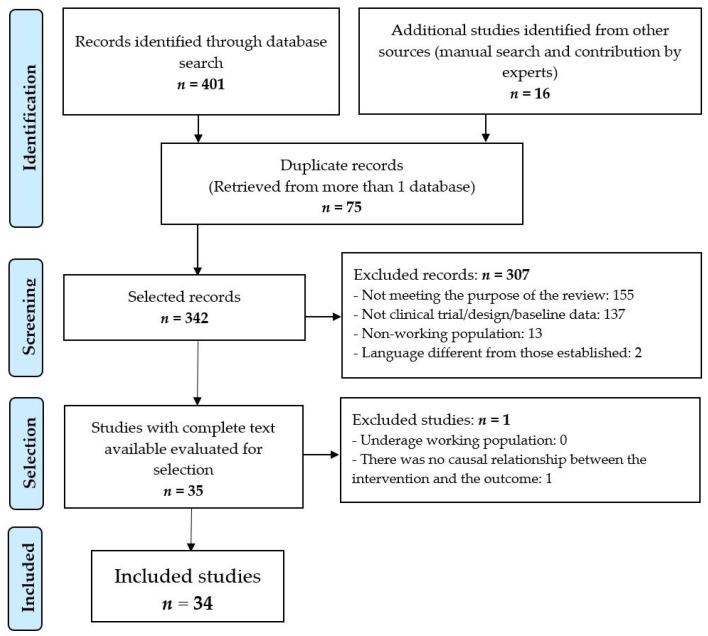
Selection procedure of the studies.

After filtering the 75 repeated records and applying the inclusion and exclusion criteria (Figure 1), 34 clinical trials [22,23,24,25,26,27,28,29,30,31,32,33,34,35,36,37,38,39,40,41,42,43,44,45,46,47,48,49,50,51,52,53,54,55] were selected for review and critical analysis (see Table 1).

The agreement among the evaluators regarding the relevance of selected studies, calculated using the KI, was 74.88% (*p* = 0.01).

The selected articles had an actuality, as determined by the BK, equal to 7.50 years, with a PI of 29.41%. The years with the highest number of published works were 2012, 2015 and 2017, with four trials published each of those years; see Table 1.

When evaluating the transparency of reporting of the trials selected for the review, the CONSORT checklist scores ranged from a minimum of 3.50 (14% compliance) to a maximum of 20.50 (82.50% compliance) with a median of 12.75 (Table 2), observing, across time, a good increasing exponential trend (R^2^ = 0.62; *p* < 0.001).

Based on the SIGN criteria, this review presented evidence with a grade of 1- (systematic review of randomized clinical trials or randomized clinical trials with a high risk of bias) with a recommendation grade of B (a body of evidence that includes studies directly applicable to the target population and that demonstrates global consistency of the results or the extrapolation of studies rated as 1).

The majority of the studies included in the review were from the USA, with 17 trials [22,25,31,34,35,36,37,39,40,42,43,45,47,48,50,54,55] and the Netherlands, with three trials [28,38,51].

The study with the largest population was that by Fernández et al. [35], with *n* = 2614 workers, and the study with the smallest population was that by Almeida et al. [36], with 28 workers. All participants were of working age (between 18 and 65 years).

The mean body mass index (BMI) in the intervention group fluctuated between a minimum of 23.8 ± 3.5 in the study by Iturriaga et al. [24] and a maximum of 39.4 ± 6.9 in the study by Barham et al. [47]. There were four clinical trials that did not report BMI: Shrivastava et al. [29] only indicated the percent of obese individuals; Follick et al. [55] only included the percent of overweight individuals; Baer [54] only reported weight in kilograms and Ferdowsian et al. [48] did not report any measure related to BMI.

The main pathologies observed in the study population were overweight and obesity [22,23,24,25,35,36,38,40,41,42,43,46,50,52]; overweight [26,29,53,55]; obesity [37,45,51]; overweight, obesity and diabetes [31,32,34,39,47,48]; abdominal obesity and dyslipidemia [27,30]; overweight and musculoskeletal disorders [28]; obesity, diabetes and cardiovascular disease [33]; metabolic syndrome [44]; metabolic disease [49], and metabolic disease [54].

The intervention period ranged from a minimum of 10 weeks [34,42] to a maximum of 3 years [50], with 12 months being the most frequent intervention period [22,26,36,37,40,41,45,46,53,54].

### 3.1. Types of Interventions Performed

Due to the heterogeneity of the actions carried out, in the clinical trials analyzed, the different interventions carried out were grouped into the following seven categories:

1. Dietary interventions associated with other actions (exercise or educational program): seven studies [23,27,30,46,48,52,53].
2. Interventions related to the workplace environment, including educational actions, financial incentives, availability and price of food and portion control: five studies [22,32,35,43,45].
3. Exclusive educational interventions aimed toward lifestyle, dietetics, physical activity, and stress management, including televigilance devices and counseling: 16 studies [25,26,28,29,33,34,36,37,40,41,42,44,47,49,51,54].
4. Economic incentives added to training actions aimed at weight loss, physical activity and dieting: three studies [31,50,55].

Multicomponent intervention, through the combination of mindfulness, e-coaching and the addition of fruits and vegetables: one study [38]; or dietary intervention (facilitating a greater supply of food in cafeterias): one study [39]; or intervention focused on physical exercise: one study [24].

### 3.2. Main Results Derived from the Interventions

From the interventions developed, the following results could be verified:

Dietary interventions associated with other actions (mainly physical exercise) decreased body weight in the intervention group [27,46,53]. Gepner et al. [30] also observed improvements in cardiometabolic markers, and Ferdowsian et al. [48], along with weight loss, reported a decrease in waist circumference. The intervention program implemented by Röhling et al. [23] (the SAMMAS intervention) achieved long-term weight loss maintenance. In contrast, in a previous trial, Leslie et al. [52] concluded that the body weight maintenance intervention was not effective.

Behavioral environmental strategies improved food selection, which, according to Thorndike et al. [22], resulted in improvements in body weight. Even educational actions were effective in promoting healthy diets [32] and were postulated as promising long-term interventions (2 years) [35]. However, Linde et al. [43] and Brehm et al. [45] indicated that environmental changes in the workplace were not enough to improve the weight and health of workers.

Educational interventions showed their suitability for implementation in the workplace; such interventions resulted in a decrease in weight and BMI in the treatment group [25,29,36,41,47]. Kempf et al. [26] also observed a decrease in BMI in the intervention group, but their results were not supported by intention-to-treat analysis. Mitchell et al. [34] confirmed that weight loss was associated with greater attendance at educational intervention sessions. Likewise, educational actions improve metabolic parameters [49], cardiometabolic risk factors [40] and the prevalence of metabolic syndrome [44]. Furthermore, this type of intervention was shown to be valid in improving the risks associated with coronary disease [54].

For the follow-up of these training activities, van Wier et al. [51] demonstrated that telephone follow-up was effective. However, Solenhill et al. [33] found that telephone counseling did not have positive effects on employees, and Thorndike et al. [42] concluded that online support was not effective.

Viester et al. [28] concluded that these actions could have promising long-term effects, but differences between the intervention and control groups were not significant. The study by Østbye et al. [37] found no differences related to the implementation of an educational program.

The use of economic incentives as the main intervention influenced the weight loss of participants [31] and even decreased attrition [55]. It was also effective in stimulating change toward healthier attitudes, reducing the tendency to increase body weight [50]. However, no study showed results related to the period after the incentive ceases.

Other multicomponent actions [38] (combination of mindfulness, e-coaching and the addition of fruits and vegetables) did not show clear causal effects at 6 and 12 weeks.

Exclusive dietary action (a low-fat vegan diet) [39] produced an improvement in body weight at 18 weeks, but results for the maintenance period and long-term results were not indicated.

For an intervention focused on physical exercise [24], beneficial effects on body composition were observed in the short term (12 weeks), but postintervention results were not included.

### 3.3. Main Results Derived of Meta-Analysis

For the meta-analysis, 35 groups of 22 articles were included.

- Effect size

**Figure 2:**
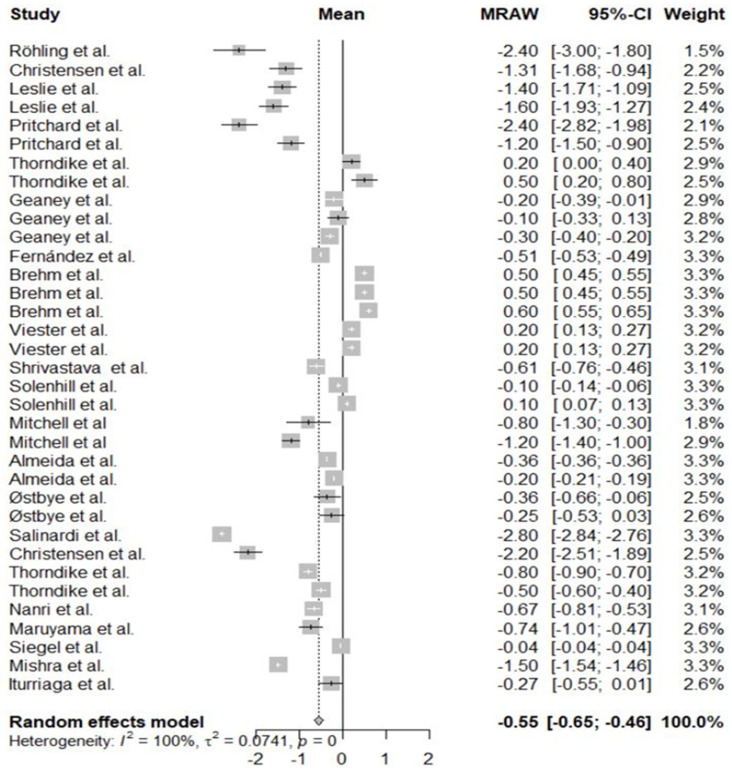
Forest plot for Body Mass Index.

The effect sizes calculated from the meta-analysis are shown in Figure 2 as well as tests for the presence of heterogeneity.

- Heterogeneity of included studies

**Figure 3:**
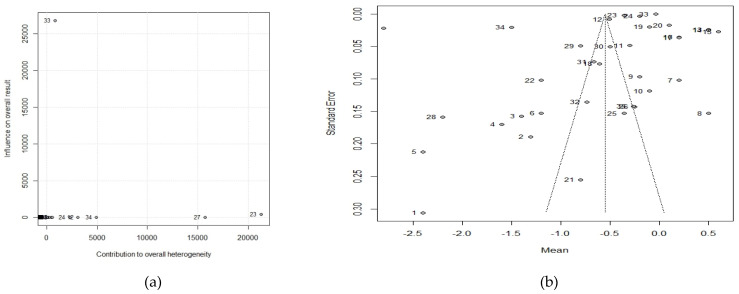
(**a**) Baujat plot for Body Mass Index (**b**) Funnel plot for Body Mass Index.

The included studies show strong heterogeneity (100%). Table 3 shows the effect of each study on the total heterogeneity. We observe that none of them is very influential. Similarly, we can see in Figure 3 of the Baujat graph that no study is in the upper right corner.

The Bajujat graph (Figure 3a) shows that no single group has a decisive weight on the outcome of the meta-analysis. In fact, in the leave-one-out test, no study varied heterogeneity by more than 1%.

- Heterogeneity of non-included studies (publication bias)

Another source of heterogeneity could come from publication bias. For this purpose, we analyze the symmetry of the Funnel-polt (Figure 3b).

As can be seen, there is not much symmetry, so publication bias may be high. The results of the Trim-fill and Copas techniques suggest that many unpublished studies, between 12 and 48 respectively, would be needed to compensate for this lack of symmetry. The model proposed by Copas would reduce the size of the effect on BMI but still be significant, in the case of the Trim-fill adjustment this reduction would end up not being statistically significant.

- Moderator analysis (meta-regression)

Other sources of possible heterogeneity may be the influence of covariates or moderators. Table 4 studies the effects of the five types of interventions and the baseline BMI.

There is variation in the effect size of BMI by baseline. Interventions give better results on obese groups than on groups with overweight or normal weight. As for the interventions, 1 and 2 were statistically significant in the multivariate model but with opposite directions. Intervention 1 decreases BMI and intervention 2 increases BMI.

## 4. Discussion

Following the recommendations regarding the objectives of a systematic review [56], the current review synthesized the relevant information related to nutrition, food and diet interventions implemented in occupational health to provide the scientific community with relevant information that can help promote new interventions for workers protection. In addition, this study is part of the strategy of the World Health Organization that emphasizes the importance of establishing primary prevention and interventions aimed at improving occupational health [57].

It could be considered that the most prevalent occupational disease (although it is not considered as such) is undoubtedly obesity (and overweight) because it affects many workers [58] and those who are overweight or obese are more likely to suffer injuries than normal-weight workers [59].

The analysis of the actuality of the reviewed studies demonstrated the full validity of the selected studies because the data obtained showed greater relevance than what was calculated for the bibliometric studies in fields related to the sciences of nutrition and occupational health [4] and more current than that found for recent systematic reviews related to occupational health [60,61].

The evaluation of the reporting transparency of the studies included in the review articles, as assessed by the CONSORT criteria, was similar to that for other review articles [62,63]. The analysis of the progression of documentary adequacy that was observed in the most current articles was mainly due to the implementation of CONSORT criteria. In fact, the oldest works did not usually follow these quality guidelines; for example, the first documents that used the CONSORT criteria date back to 1996 [64], and their use was progressive. If clinical trials have an inadequate methodology or, especially, if the final description of the trial does not contain certain information, readers cannot adequately judge the validity of the study, and the scientific evidence related to the results is very limited [65].

The level of evidence and grade of recommendation for this study, as determined using the SIGN criteria, were consistent or even better than those observed in previous studies. Despite seeking a consistent cause-effect relationship, because intervention studies were sought, some were subject to more bias than others and therefore, more weakly support the conclusions [66]. The conclusions of many studies of occupational health and safety are still not based on the greatest possible evidence [67]. This may be due to the experimental design of primary studies, such as clinical trials, which are considered robust but may not be adequate to evaluate interventions in occupational health when presenting, generally, very long-term effects; furthermore, as in this review, nutritional interventions were not the most studied mediations in relation to work and were more oriented to combat certain diseases.

All the studies focused primarily on overweight and/or obese populations, except for the studies by Nanri et al. [44], which focused on a population with metabolic syndrome, that by Maruyama et al. [49], which focused on metabolic disease in general and that by Baer [54], which focused on heart disease.

BMI was provided in the vast majority of the studies, and it was considered that those that did not provide a clear measure to evaluate interventions [29,48,54,55] could not have adequately reported their results. This inadequate description of clinical studies can be, in any case, a waste of time for those who seek valid information derived from clinical trials [65]. The lack of information in a publication can result in the work being excluded when carrying out a systematic review on a certain intervention. Approximately one-third of clinical trials can be excluded from systematic reviews because relevant data are lacking [68]. In this review, it was decided to retain these four clinical trials to provide as much information as possible but not dismiss the lack of relevant results.

Dietary-nutritional interventions within companies are always complex due to the idiosyncrasy of the workforce and, generally, the short period available to perform these interventions [69]. Thus, the follow-up period must be adequate to assess the results of the intervention, a requirement that all the selected trials met. A period of several weeks, even months, is considered necessary to assess the results [61,70].

In general, interventions using any mode of interaction (face-to-face, telephone, internet, etc.), directed by a trained professional, were effective in improving outcomes related to overweight and obesity.

From the interventions observed, it was possible to deduce that the actions that included several strategies achieved adequate results in the working population. This statement is consistent with the results reported by Upadhyaya et al. [71], who concluded that occupational health professionals should continue to be creative in the development of multicomponent interventions (combining behavioral/educational, environmental and organizational support).

The effectiveness of dietary interventions associated with other actions (mainly physical exercise) is a well-known topic. Their effectiveness in the management of obesity and overweight in the work environment has already been demonstrated [72,73]. However, the structures and cultures of the workplace should always be considered when planning interventions. The negotiation and flexibility of stakeholders play essential roles in overcoming resistance to change [74].

Among the combined strategies, environmental interventions have been proposed as appropriate actions for the promotion of healthy habits, although they were not considered sufficient, by themselves, to improve the weight and health of workers [43,45]. Thus, the review by Chu et al. [75], confirmed the consistency of the effectiveness of multicomponent environmental interventions.

The results obtained showed a causal relationship when implementing educational measures in the workplace focused on decreasing body weight, resulting in improvements in metabolic parameters [49], cardiometabolic risk factors [40] and prevalence of metabolic syndrome [44]. This type of intervention was shown to be valid in improving the risks associated with coronary disease [54]. However, the review by Wolfenden et al. [76] concluded that it was not clear whether such strategies were profitable or generated unintended adverse consequences, thus justifying more research to seek more evidence.

The strategies that included financial incentives (generally discounts for healthy items on the menu for the company cafeteria) when choosing the healthiest menu items were shown to be effective in preventing obesity and improving eating habits. However, the study by Sawada et al. [77] expressed the need to carry out interventions that focus exclusively on financial incentives versus no intervention to determine if this strategy has a clear impact. Combined actions could mask these results.

In line with what was stated by Peñalvo et al. [78], it is important to highlight the generally short/moderate duration (between 6 months and 1 year) of the vast majority of workplace health interventions and programs, as well as the limited evaluation of the sustainability of the change in habits after the end of the program, which may raise doubts about the long-term effectiveness of these interventions. However, in relation to the failure of interventions focused on overweight and obese patients carried out in the workplace, Park and King [72] argue that there is evidence indicating that the duration of the intervention is a determining factor, with short-term programs (less than 6 months) being more effective than long-term programs.

Most of the identified studies came from high-income countries, mainly the United States, where the problem of obesity and overweight has become a heavy burden in economic and health terms for the state and companies [7]. In this sense, and as indicated by Peñalvo et al. [78], occupational health programs and their evaluation are scarce in other geographic and socioeconomic contexts (a single study from India included in the review) where non-communicable diseases are increasing rapidly.

In short, given the substantial period of time that adults spend in their workplaces each day, workplaces provide an opportune environment for interventions relating to healthy habits and can be effective if such interventions combine several strategies (diet, lifestyle, physical activity, reduction in alcohol and tobacco consumption, rewards, adherence to the intervention, etc.) The identification of strategies that are effective in improving the implementation of interventions in the workplace has the potential to improve health outcomes.

However, from the results observed in the clinical trials reviewed, employees acquired a greater awareness of the relationship between diet and health. Additionally, they considered the actions taken a positive experience for themselves and the company. These statements had already been noted in a previous study by Munar-Gelabert et al. [69].

The results of the meta-regression and the little-observed effect derived from the interventions are in line with other previous works. The findings of LaCaille et al. [79], showed that ecological approaches in the workplace have had little or no effect on preventing weight gain. Similarly, Allan et al. [80], in a 2017 systematic review, noted that there was no convincing evidence that this type of intervention resulted in weight or BMI changes. Another limitation of environmental interventions is the cost and levels of administrative approval necessary for modifying the work environment since they can pose a barrier to the implementation and success of environmental strategies. In addition, there may be reluctance regarding healthy alternatives available in the cafeteria and portion size reduction among workers [79]. Moreover, Vermeer et al. [81], noted the importance of assessing the existence of workers’ compensatory eating behaviors after eating less in the workplace.

### 4.1. Limitations of the Review

The results of this review are limited by the shortcomings of each work included in it. The level of evidence and recommendation values reached did not ensure that the clinical trials reviewed did not have a high risk of bias. Numerous studies did not specify whether they controlled for confounding factors that could affect the results.

In addition, to raise the level of evidence and recommendation of this review, it would be necessary for all the trials to have taken into account the existence of adverse consequences, an item not observed in any of the included studies. Thus, the low-certainty evidence suggests that such strategies can make little or no difference in the measures of the consistency of implementation or in the different health behavior outcomes of the employees, a circumstance already reported by Wolfenden et al. [76].

### 4.2. Critical Analysis of the Authors

While the majority of clinical trials found that the different interventions observed provided opportunities to establish different programs in the workplace, other studies contradicted this possibility by not finding an association between the intervention group and the control group. Additionally, and without doubting the favorable results obtained, many of the trials did not report effects since the intervention ended.

It would have been desirable to have considered the impact that shift work had when implementing the different interventions. This issue was not clear in the documents reviewed.

Another issue that was missed was the absence of information on adherence to the different interventions. As stated by Abbate et al. [82], the follow-up of any strategy is fundamental because it is directly related to health outcomes.

From the meta-regression study, it was observed that the interventions give better results in people who presented high BMI values (obesity). In contrast, intervention 2 (interventions related to workplace environment) would not give the expected results (it would increase the BMI). In addition, although the characteristics of the workplace can generate an obesogenic environment, changes in this environment may be necessary but not enough to modify the obesity-related health behaviors of workers.

Importantly, methodologically rigorous studies are considered necessary to carry out adequate nutritional interventions in the workplace.

## 5. Conclusions

Given that most people spend a large part of their time in the workplace and, therefore, eat at least one of their daily meals there, well-planned interventions—preferably including several strategies—have been shown to be useful for reducing weight, improving healthy behaviors and preventing overweight and obesity.

## Figures and Tables

**Table 1:** Summary of the studies reviewed.

Author, Year	Population Studied	Pathology	Country	Intervention Period	Type of Intervention	Observed Outcome
Thorndike et al., 2021 [22]	N = 602 Massachusetts General Hospital employees. IG: *n* = 299 M/F = 69/230 Age Mean ± SD = 43.5 ± 12 BMI ± SD = 28.6 ± 6.6 CG: *n* = 303 M/F = 55/248 Age Mean ± SD = 43.8 ± 12.5 BMI ± SD = 28.0 ± 6.5	Overweight and obesity	USA	12 months	IG: Participants received two emails per week with feedback on previous cafeteria purchases and personalized health and lifestyle tips and one letter per month with peer comparisons and financial incentives for healthier purchases. Emails and letters were automatically generated using survey, health, and cafeteria data. CG: Participants received one letter per month with general healthy lifestyle information.	There were no between-group differences in weight change at 12/24 months. The IG increased green-labeled purchases and decreased red-labeled and calories purchased compared with CG (*p* < 0.001) at 12/24 months. The findings suggest that an automated behavioral intervention using workplace cafeteria data improved employees’ food choices but did not prevent weight gain.
Röhling et al., 2020 [23]	N = 30 Düsseldorf Catholic Hospital employees IG: Starting intervention (SI): *n* = 15 M/F = 3/12 Age Mean ± SD = 44 ± 9 BMI ± SD = 35.1 ± 6.9 CG: Waiting list (WL): *n* = 15 M/F = 2/13 Age Mean ± SD = 49 ± 7 BMI ± SD = 32.8 ± 6.1	Overweight and obesity	Germany	12 weeks	All participants were equipped with telemetric devices (scales and pedometers). IG: Immediately started with SAMMAS intervention (group-based seminars, low-carbohydrate nutrition including formula diet, continuous glucose monitoring, telemetric monitoring, and telemedical coaching) with weekly contacts. CG: Continued their habitual lifestyle. At 12 weeks, they started the same SAMMAS intervention.	SI-group significantly reduced weight (*p* < 0.001) and improved in BMI, WC, fat mass, and all variables of eating behavior (all *p* < 0.05) compared to the WL-group after 12 weeks of intervention. The Low-Insulin-Method included in the multi-component, occupational healthcare program SAMMAS could be an effective and promising new approach for the reduction in body weight and long-term weight loss maintenance in people with overweight and obesity.
Iturriaga et al., 2019 [24]	N = 63 (workers not described) IG: *n* = 34 M/F = 0/34 Age Mean ± SD = 42.53 ± 5.34 BMI ± SD = 23.78 ± 3.52 CG: *n* = 29 M/F = 0/29 Age Mean ± SD = 45.01 ± 4.93 BMI ± SD = 25.64 ± 5.12	Overweight and obesity	Spain	12 weeks	IG: A moderate-intensity aerobic physical exercise program consisting of two different activities, Zumba or Aqua fitness. Three sessions per week (36 sessions in total) of duration 45 min per session.	Beneficial effects on body composition of a short-term workplace aerobic exercise program (12 weeks) were observed in terms of reduced BMI (*p* = 0.004), fat indexes, and fat mass (*p* = 0.001) in the lower limbs compared to controls.
Day et al., 2019 [25]	N = 421 firefighters IG: *n* = 217 M/F = 168/49 Age Mean ± SD = 37.3 ± 12.7 BMI (%), (BMI < 25) = 27.4 (BMI 25–29.9) = 32.1 (BMI ≥ 30) = 40.5 CG: *n* = 178 M/F = 149/29 Age Mean ± SD = 36.9 ± 12.6 BMI (%), (BMI < 25) = 16.3 (BMI 25–29.9) = 32.6 (BMI ≥ 30) = 51.1	Overweight and obesity	USA	6 months	IG: They were provided TF20 (First Twenty Intervention) web-based program and information about enrollment and use. The TF20 includes modules on physical activity, nutrition and behavioral health to provide 24 weeks of evidence-based material. With weekly goals, messages vía emails, tasks, resources and tracking tools. CG: Usual wellness practices. They received the same introduction, program overview, follow-up and evaluation as the IG.	All models indicated an average weight gain for the control group and weight loss for the treatment group. The treatment effect in the measured weight of all participants and those overweight and obese approached statistical significance, *p* = 0.08 and *p* = 0.07, respectively, despite the relatively small samples. TF20 supports firefighters’ weight loss.
Kempf et al., 2019 [26]	N = 104 Boehringer Ingelheim workers IG Telemedical coaching: *n* = 34 M/F = 29/5 Age Mean ± SD = 51 ± 6 BMI ± SD = 32 ± 7 CG1: *n* = 34 M/F = 25/5 Age Mean ± SD = 48 ± 5 BMI ± SD = 30 ± 4 CG2: *n* = 36 M/F = 30/6 Age Mean ± SD = 51 ± 5 BMI ± SD = 31 ± 4	Overweight	Germany	12 months	TMC and CG1 were equipped with tele monitoring devices (scales and pedometers) at baseline and CG2 after 6 months. All participants were instructed to monitor their body weight and physical activity. IG: (TMC): Was coached with weekly care calls in months 3–6 and monthly calls from months 7 to 12. CG1: Received no further support. CG2: Had a short coaching phase in months 6–9.	All groups reduced weight after 12 months (*p* < 0.01) and sustained it during follow-up (*p* < 0.01). All groups reduced BMI, systolic and diastolic blood pressure and improved eating behavior. TMC and/or tele monitoring support long-term weight reduction in overweight employees. The combination of both interventions points towards an additional effect (not supported by the intention to treat analysis).
Tene et al., 2018 [27]	N = 277 research center workers G1 (Low-Fat Diet): *n* = 139 M/F: 122/17 Age Mean ± SD = 48.4 ± 9.2 BMI ± SD = 30.8 ± 3.7 G2 (Mediterranean/Low-Carbohydrate Diet): *n* = 138 M/F: 124/14 Age Mean ± SD = 47.5 ± 9.3 BMI ± SD = 30.9 ± 3.9 At 6 months of DI randomized groups with physical activity for the last 12 months of intervention: G1: LF ^PA+^/MED-LC ^PA+^ G2 LF ^PA−^/MED-LC ^PA−^	Abdominal obesity or dyslipidemia	Israel	18 months	Dietary intervention (DI): 1500 kcal/day for women and 1800 for men (both diets), included weekly nutritional sessions. -Mediterranean/low-carbohydrate diet: Provided 28 gr walnuts/day. -Low Fat diet: limit total fat intake to 30% of calories, up to 10% of saturated fat, and no more than 300 mg of cholesterol per day, and to increase dietary fibers. Physical activity (PA) intervention: Free 12-month gym membership, and monthly 60-min educational workshops in the workplace.	At 6 months pancreatic-fat significantly decreased (*p* = 0.002), similarly between diets (*p* = 0.736) and after 18 months (*p* = 0.049). This study shows modulation of pancreatic fat through lifestyle interventions, mainly by increasing dietary fat proportion on the account of the relative carbohydrate intake. The efficacy is best achieved when accompanied by moderate endurance exercise.
Viester et al., 2017 [28]	N = 314 Construction Workers IG: *n* = 162 M/F: 162/0 Age Mean ± SD = 46.3 ± 9.9 BMI ± SD = 27.3 ± 3.5 CG: *n* = 152 M/F: 152/0 Age Mean ± SD = 47.0 ± 9.5 BMI ± SD = 27.4 ± 3.9	Overweight and musculoskeletal disorders	Netherlands	6 months	IG: They received individual coaching sessions, tailored information, and materials to improve lifestyle behavior (Physical activity and dietary behavior). CG: Received usual care.	Positive changes showed in vigorous physical activity and intake of sugar-sweetened beverages compared to controls, as well as effects on body weight (*p* = 0.010), BMI (*p* = 0.010), and waist circumference (*p* = 0.032) at 6 months. Long-term effects were still promising but not statistically significant.
Shrivastava et al., 2017 [29]	N = 267 corporative workers IG: *n* = 156 M/F: 137/19 Age Mean ± SD = 35.8 ± 7.6 BMI ± SD = 28.21 ± 2.89 CG: *n* = 111 M/F: 92/19 Age Mean ± SD = 39.0 ± 8.7 BMI ± SD = 28.20 ± 3.59	Overweight	India	6 months	IG: A multicomponent intervention with sessions on the different topics related to healthy living, diet, stress and physical activity. And monitoring the compliance of lifestyle changes with digital resources. CG: No intervention, but were given general health talk twice in six months.	Active intervention was successful in achieving of reduction in weight, excess subcutaneous fat, and cardiometabolic risk factors after 6 months. Statistically significant changes in IG vs. CG in weight, BMI, waist circumference, hip circumference (*p* < 0.001).
Gepner et al., 2017 [30]	N = 278 research center workers G1 (Low-Fat Diet) *n* = 139 M/F: 122/17 Age Mean ± SD = 48.4 ± 9.2 BMI ± SD = 30.8 ± 3.7 G2 (Mediterranean/Low-Carbohydrate Diet): *n* = 139 M/F: 125/14 Age Mean ± SD = 47.4 ± 9.3 BMI ± SD = 30.9 ± 4.0 At 6 months of DI randomized groups with physical activity for the last 12 months of intervention: G1: LF ^PA+^ /MED-LC ^PA+^ *n* = 126 G2 LF ^PA−^/MED-LC ^PA−^ *n* = 130	Abdominal obesity or dyslipidemia	Israel	18 months	Dietary intervention (DI): Monitored provided lunch (1500 kcal/day women and 1800 men) and a 90-min nutritional session in the workplace with clinical dietitians every week (first month), and every month thereafter. -Mediterranean/low-carbohydrate diet: Provided 28 g walnuts/day. -Low Fat diet: limit total fat intake to 30% of calories, up to 10% of saturated fat, and no more than 300 mg of cholesterol per day, and to increase dietary fibers. Physical Activity Intervention: Free supervised gym membership for 12 months, three sessions per week, included monthly 60-min educational workshops.	Energy intake decreased similarly across diet groups after 6 months (*p* = 0.85) and 18 months (*p* = 0.18), and all were significantly lower compared with baseline (*p* < 0.001). A Mediterranean diet, rich in unsaturated fats and low in carbohydrates, and being physically active can improve cardiometabolic risk markers through changes in visceral/ectopic fat depots that are not reflected by mild body weight changes alone.
Faghri et al., 2017 [31]	N = 99 nursing-home employees M/F: 9/88 Age Mean ± SD = 46.98 ± 11.36 BMI ± SD = 35.33 ± 6.91 IG: Incentivized participants (IP): *n* = 51 CG: Non incentivized participants (NIP): *n* = 48	Overweight, obesity and diabetes	USA	16 weeks	IG: Financial incentive-based intervention. All participants received a personalized weight-loss consultation based on their reported physical activity habits and dietary preferences. Each participant received an action plan based on the National Diabetes Prevention Program (NDPP) CG: No incentive.	IP reduced more weight (*p* = 0.027) and BMI (*p* = 0.043) than NIP at week 16. At week 28, IP lost more weight than NIP (*p* = 0.053), and reduced their BMI more than NIP (*p* = 0.308). Eating and exercise self-efficacy were significant mediators between health behaviors and weight loss (*p* < 0.05). Incentives significantly moderated the effects of self-efficacy (*p* = 0.00) on weight loss. Self-efficacy and financial incentives may affect weight loss and play a role in weight-loss interventions.
Geaney et al., 2016 [32]	N = 541 manufacturing workplaces IG1 (NE): *n* = 107 M/F = 81/26 Age *n* (%) 18–29 = 13 (12.1) 30–44 = 67 (62.6) 45–65 = 27 (25.2) BMI ± SD = 27.1 ± 4.1 IG2 (EDM): *n* = 71 M/F = 43/28 Age *n* (%) 18–29 = 7 (9.9) 30–44 = 33 (46.5) 45–65 = 31 (43.7) BMI ± SD = 28.0 ± 5.1 IG3 (NE + EDM): *n* = 272 M/F = 227/45 Age *n* (%) 18–29 = 13 (4.8) 30–44 = 197 (72.4) 45–65 = 62 (22.8) BMI ± SD = 27.1 ± 3.8 CG: *n* = 67 M/F = 42/25 Age *n* (%) 18–29 = 11 (16.4) 30–44 = 34 (50.7) 45–65 = 22 (32.8) BMI ± SD = 27.6 ± 4.2	Obesity and type 2 diabetes	Ireland	7–9 months	Nutrition education (NE) was comprised of three elements: monthly group nutrition presentations, detailed group nutrition information (daily traffic light menu labeling and monthly posters, leaflets and emails) and individual nutrition consultations. Environmental dietary modification (EDM) included five elements: (a) menu modification: restriction of saturated fat, sugar and salt, (b) increase in fiber, fruit and vegetables, (c) price discounts for whole fresh fruit, (d) strategic positioning of healthier alternatives and (e) portion size control.	Effects in the education and environment alone workplaces were smaller and generally non-significant. There were significant positive changes in intakes of saturated fat (*p* = 0.013), salt (*p* = 0.010), nutrition knowledge (*p* = 0.034) and BMI (*p* = 0.047) between baseline and follow-up in the combined intervention versus the control. Combining nutrition education and environmental dietary modification may be an effective approach for promoting a healthy diet and weight loss at work.
Solenhill et al., 2016 [33]	N = 981 transportation companies employees M/F: 655/326 Age Mean ± SD = 44 ± 10.2 IG1 (intervention Web): *n* = 301 BMI ± SD = 26.6 ± 4.4 IG2 (intervention Web + telephone): *n* = 324 BMI ± SD = 26.1 ± 4.0 CG: *n* = 356 BMI ± SD = 26.6 ± 4.5	Obesity, diabetes, and cardiovascular diseases	Sweden	9 months	IG1: They received tailored Web-based health feedback. IG2: They received tailored Web-based health feedback + additional optional telephone health coaching for those participants who were motivated to change health behaviors. CG: No additional intervention.	Tailored Web-based health feedback and the offering of optional telephone coaching did not have a positive health effect on employees in the transport services.
Mitchell et al., 2015 [34]	N = 254 Latino farmworkers M/F: 71/183 IG: *n* = 174 Age Mean ± SD = 32.3 ± 7.6 BMI ± SD = 29.1 ± 0.3 CG: *n* = 80 Age Mean ± SD = 32.5 ± 7.9 BMI ± SD = 27.7 ± 0.4	Overweight, obesity and diabetes	USA	10 weeks	IG: 10 weekly educational sessions (health habits, physical activity and dietary behaviors) led by promoters. CG: No intervention.	Greater losses in weight (*p* = 0.0002), BMI (*p* = 0.0001), and waist circumference (*p* = 0.001) were associated with increasing attendance at intervention sessions. Women significantly reduced weight (*p* = 0.001) and BMI (*p* = 0.002) compared with controls, except blood glucose. The successful pilot workplace intervention offers a model to reach otherwise difficult-to-access Latino farmworkers.
Fernández et al., 2015 [35]	N = 2614 manufacturing, research, and development company employees IG: *n* = 1547 M/F: 1054/493 Age Mean ± SD = 47.7 ± 7.47 BMI ± SD = 28.6 ± 5.50 CG: *n* = 1067 M/F: 594/474 Age Mean ± SD = 47.4 ± 7.84 BMI ± SD = 28.6 ± 5.55	Overweight and obesity	USA	2 years	Environmental intervention in the worksite. Employees received a small economic incentive. The intervention promotes healthy lifestyles through portion control, education, healthy diets, and physical activity.	BMI decreased significantly at the intervention worksites after 2 years (*p* = 0.03) and non-significantly at the control worksites (*p* = 0.6). Worksite environmental interventions may be promising strategies for addressing weight control at the population level.
Almeida et al., 2015 [36]	28 worksites (workers not described) N = 1790 employees IG INCENT: *n* = 789 M/F (% ± SD) = 19.79/80.21 ± 10.84 Age Mean ± SD = 45.68 ± 3.30 BMI ± SD = 33.26 ± 6.39 IG LMW: *n* = 1001 M/F (% ± SD) = 32.57/67.43 ± 25.02 Age Mean ± SD = 48.24 ± 2.78 BMI ± SD = 33.51 ± 6.44	Overweight, and obesity	USA	12 months	Two weight loss interventions targeted diet and physical activity behaviors: IG INCENT: Individually targeted Internet-based intervention with monetary incentives. INCENT was delivered via daily e-mails over 12 months. IG Livin’ My Weigh (LMW): a less-intensive minimal intervention that included newsletters and onsite educational sessions delivered on a quarterly basis. LMW was delivered quarterly via both newsletters and onsite educational sessions.	Participants in the INCENT group on average lost 2.27 lbs (*p* < 0.001) and had a BMI decrease of 0.36 kg/m^2^ (*p* < 0.001) while participants in LMW group lost 1.30 lbs (*p* < 0.05) and decreased BMI by 0.20 kg/m^2^ (*p* < 0.05). However, the differences between INCENT and LMW groups in weight loss and BMI reduction were not significant. Both approaches investigated were successful in helping participants lose small amounts of weight and decrease their BMI.
Østbye et al., 2015 [37]	N = 550 Duke University and Medical Center employees IG1 (WM+ behavioral): *n* = 275 M/F: 45/230 Age: < 35 = 42 35–50 = 133 >50 = 100 BMI ± SD = 37.37 ± 6.61 IG2 (WM educational): *n* = 275 M/F: 48/227 Age: <35 = 53 35–50= 134 >50 = 88 BMI ± SD = 37.02 ± 6.14 Used in the analysis WM (*n* = 220) WM+ (*n* = 215)	Obesity	USA	1 year	Weight Management [WM]: Educational program targeting healthy lifestyle changes for weight loss (portion control, education, healthy diets, and physical activity). Weight Management Plus [WM+]: Intensive behavioral intervention: (1) monthly counseling sessions, (2) meetings with an exercise physiologist (3) quarterly biometric feedback, (4) targeted health education materials, and (5) information and active linking with various Duke programs and wellness resources, (6) use of eHealth trackers for diet and weight.	There were no clinically, or statistically, meaningful differences between groups but there were modest reductions in body mass index and positive, meaningful changes in diet and physical activity for both groups.
Van Berkel et al., 2014 [38]	N = 257 research institutes employees IG: *n* = 129 M/F: 47/82 Age Mean ± SD = 46.0 ± 9.4 BMI ± SD = 24.74 ± 3.96 CG: *n* = 128 M/F: 37/91 Age Mean ± SD = 45.1 ± 9.6 BMI ± SD = 24.66 ± 3.56	Overweight and obesity	Netherlands	6 months	IG: 8 weeks of in-company mindfulness training with homework exercises, followed by eight sessions of e-coaching (in their free time). Additionally, free fruit and snack vegetables were provided for 6 months. CG: They received information on existing lifestyle behavior-related facilities that were already available at the worksite.	This study did not show an effect of a worksite mindfulness-based multi-component intervention on lifestyle behaviors and behavioral determinants after 6 and 12 months.
Mishra et al., 2013 [39]	N = 291 GEICO corporate offices employees IG: *n* = 142 M/F: 32/110 Age Mean ± SD = 44.3 ± 15.3 BMI ± SE = 34.7 ± 0.6 CG: *n* = 149 M/F: 18/131 Age Mean ± SD = 46.1 ± 13.6 BMI ± SE = 35.3 ± 0.7	Overweight, obesity and type 2 diabetes	USA	18 weeks	IG: a low-fat vegan diet, with weekly group support and work cafeteria options available plus a daily supplement of vitamin B12. CG: No Intervention. They were given $50 gift certificates for completion of all aspects of the study.	An 18-week dietary intervention using a low-fat plant-based diet in a corporate setting improves body weight (*p* < 0.001), BMI (*p* < 0.001), plasma lipids (*p* = 0.001), and, in individuals with diabetes, glycemic control (*p* = 0.003).
Salinardi et al., 2013 [40]	N = 466 Boston companies employees IG: *n* = 84 M/F: 21/63 Age Mean ± SD = 48.6 ± 1.2 BMI ± SD = 33.3 ± 0.7 CG: *n* = 34 M/F: 8/26 Age Mean ± SD = 49.9 ± 2.1 BMI ± SD = 33.3 ± 1.2	Overweight and obesity	USA	12 months	IG: Intervention combined recommendations to consume a reduced-energy, low-glycemic load, high-fiber diet with behavioral change education. Employees who completed the weight-loss program were invited to reenroll in the 6-mo program (identical to the original except that the groups met once per month). CG: Wait-listed weight-loss program.	Worksites can be successful locations for the implementation of interventions that cause substantial mean weight loss (*p* < 0.001) and improve cardiometabolic risk factors (total cholesterol, glucose, systolic blood pressure, and diastolic blood pressure, *p* ≤ 0.02 for each).
Christensen et al., 2012 [41]	N = 98 health care workers M/F: 0/98 IG: *n* = 54 M/F: 0/54 Age Mean ± SD = 45.7 ± 8.7 BMI ± SD = 30.7 ± 5.4 CG: *n* = 44 M/F: 0/44 Age Mean ± SD = 46.0 ± 8.6 BMI ± SD = 30.4 ± 4.9	Overweight and obesity	Denmark	12 months	IG: one-hour weekly workplace intervention consisting of diet, physical exercise and cognitive-behavioral training. CG: monthly two-hour oral presentation during working hours about the Danish Dietary recommendations and other health-related topics.	The intervention generated substantial reductions in body weight (*p* < 0.001), BMI (*p* < 0.001) and body fat percentage (*p* < 0.001). The positive results support the workplace as an efficient arena for weight loss among overweight females.
Thorndike et al., 2012 [42]	N = 330 Massachusetts General Hospital employees IG: *n* = 174 M/F: 17/157 Age Mean ± SD = 44.2 ± 11.8 BMI ± SD = 28.0 ± 5.8 CG: *n* = 156 M/F: 28/128 Age Mean ± SD = 41.6 ± 13.6 BMI ± SD = 27.5 ± 5.9	Overweight and obesity	USA	10 weeks	Ten-week exercise and nutrition program (IG and CG) immediately following by 9-month maintenance intervention. IG: Internet support with a website for goal-setting and self-monitoring of weight and exercise plus minimal personal support (for 9 months). CG: usual care (for 9 months).	The initial program resulted in moderate weight loss and improvements in diet and exercise behaviors at 1 year (*p* < 0.001) in both groups, but no difference in weight loss between groups. The Internet-based maintenance program immediately following did not improve these outcomes.
Linde et al., 2012 [43]	N = 1672 in six worksites IG: *n* = 723 M/F: 273/450 Age (%), <30 =18.3% 31–40 = 24.3% 41–50 = 31.1% 51–60 = 23.4% >60 = 3.0% BMI ± SD = 28.7 ± 6.6 CG: *n* = 949 M/F: 40.5%/59.5% Age (%), <30 =15.6% 31–40 = 26.0% 41–50 = 31.9% 51–60 = 22.8% >60 = 3.7% BMI ± SD = 28.3 ± 6.1	Overweight and obesity	USA	2 years	IG: A four-component environmental intervention focused on food availability and price, physical activity promotion, scale access, and media enhancements to promote a healthier workforce and improve weight control. CG: Following the last round of data collection, control sites were offered a DVD containing intervention materials and an opportunity to ask questions of intervention staff as needed.	BMI was not significantly affected by environmental changes. Mean weight and BMI gain was higher at intervention sites relative to controls. Results about environmental change at worksites may be not sufficient for population weight gain prevention.
Nanri et al., 2012 [44]	N = 102 employees of a company in Kanagawa Prefecture IG: *n* = 49 M/F: 49/0 Age Mean ± SD = 53.7 ± 6.1 BMI ± SD = 26.0 ± 2.4 CG: *n* = 53 M/F: 53/0 Age Mean ± SD = 52.8 ± 7.4 BMI ± SD = 25.6 ± 2.3	Metabolic syndrome (MS)	Japan	6 months	IG: received a six-month lifestyle modification program focused on exercise and diet behavior from a trained occupational health nurse at the baseline and at one and three months. CG: Standard health guidance by an occupational health nurse using a leaflet at the baseline.	The program did not lead to a greater decrease in the prevalence of metabolic syndrome. However, WC (*p* = 0.02), body weight (*p* < 0.001), BMI (*p* = 0.001) and glycated hemoglobin (*p* = 0.005) were significantly decreased in the intervention group, as well as a significant reduction in sugar and sweetener intake (*p* = 0.002), in cereal intake (*p* = 0.002) and an increase in physical activity (*p* < 0.001).
Brehm et al., 2011 [45]	N = 341 manufacturing companies employees M/F (%) = 60/40 Age Mean ± SD = 43.8 ± 10.0 BMI ± SD = 29.0 ± 5.5 IG: *n* = 168 CG: *n* = 173	Obesity	USA	1 year	IG: Multicomponent environmental intervention that included employee advisory committees, point-of-decision prompts, walking paths, cafeteria/vending changes, and educational materials.	There were no intervention effects for outcome variables. Findings indicate that subtle environmental changes alone may not impact employees’ weight and health.
Christensen et al., 2011 [46]	N = 144 health care workers IG: *n* = 54 M/F: 0/54 Age Mean ± SD = 45.7 ± 8.7 BMI ± SD = 30.5 ± 5.4 CG: *n* = 44 M/F: 0/44 Age Mean ± SD = 46.0 ± 8.6 BMI ± SD = 30.4 ± 4.9	Overweight and obesity	Denmark	12 months	IG: An individually dietary plan with an energy deficit of 1200 kcal/day, strengthening exercises and cognitive-behavioral training during working hours 1 h/week. Leisure time aerobic fitness was planned for 2 h/week. CG: Monthly oral presentations.	The significantly reduced body weight, body fat, waist circumference and blood pressure as well as increased aerobic fitness in the intervention group (*p* ≤ 0.001) show the great potential of workplace health promotion among this high-risk workgroup.
Barham et al., 2011 [47]	N = 45 employees of Onondaga County Department of Probation, Health and Social Services IG: *n* = 21 M/F: 4/17 Age Mean ± SD = 51.1 ± 9.6 BMI ± SD = 39.4 ± 6.9 CG: *n* = 24 M/F: 3/21 Age Mean ± SD = 51.2 ± 6.4 BMI ± SD = 36 ± 6.9	Overweight, obesity and type 2 diabetes	USA	3 months	IG: 3-month program (12 one-hour weekly midday group sessions) that targeted healthy diet, physical activity, and stress reduction, followed by a monthly maintenance program with the groups choosing topics that they considered of greatest benefit. CG: Wait list control group.	The IG lost significant weight compared to the wait CG over the first 3 months, with a decrease in BMI (*p* < 0.001) and waist circumference (*p* = 0.004), an increase in physical activity (*p* = 0.011) and lower dietary fat intake (*p* = 0.018). A worksite intervention program can help government employees adopt healthier lifestyles and achieve modest weight loss.
Ferdowsian et al., 2010 [48]	N = 113 GEICO company employees IG: *n* = 68 M/F: 18/50 Age Mean ± SD = 46 ± 10 BMI ± SD = Not provided CG: *n* = 45 M/F: 2/43 Age Mean ± SD = 42 ± 10 BMI ± SD = Not provided	Overweight, obesity and diabetes type 2	USA	22 weeks	IG: Follow a low-fat vegan diet for 22 weeks, group meetings, cooking demonstrations. Also provided with practical tools and a grocery store tour. CG: They were compensated with gift certificates ($60) and informed that the nutrition program would be provided upon study completion.	IG participants experienced greater weight changes compared with CG (*p* < 0.0001), as well as greater changes in waist circumference and waist ratio hip (*p* < 0.0001). An intervention using a low-fat, vegan diet effectively reduced body weight and waist circumference.
Maruyama et al., 2010 [49]	N = 99 office workers of the Nichirei Group Corporation IG: *n* = 52 M/F: 52/0 Age Mean ± SD = 43.1 ± 7.7 BMI ± SD = 25.7 ± 3.7 CG: *n* = 47 M/F: 47/0 Age Mean ± SD = 35.5 ± 8.1 BMI ± SD = 25.8 ± 3.3	Metabolic diseases	Japan	4 months	IG: Individualized assessment and collaborative goal-setting sessions based on food group intake and physical activity, followed by two individual counseling sessions with a registered dietitian and physical trainer, and received monthly website advice. CG: No intervention.	Mean inter-group differences in changes were significant at level *p* ≤ 0.01 for body weight, BMI and homeostasis model assessment of insulin resistance. And at level *p* ≤ 0.05 for fasting plasma glucose and hemoglobin A1c. The LiSM10! program improved insulin resistance-related metabolic parameters.
Siegel et al., 2010 [50]	N = 413 elementary school personnel IG: *n* = 211 M/F: 35/176 Age Mean ± SE = 40.0 ± 0.73 BMI ± SE = 28.4 ± 0.45 CG: *n* = 202 M/F: 53/149 Age Mean ± SE = 39.5 ± 0.84 BMI ± SE = 27.9 ± 0.51	Overweight and obesity	USA	3 years	IG: Develop and implement health promotion activities (improving diet, increasing physical activity, stress management, etc.) for employees. Each intervention school was given a stipend of $3500 per year (for 3 years) to subsidize its wellness activities. CG: Was given an unrestricted stipend of $1000 at baseline and follow-up.	Intervention schools presented a significant change in BMI (*p* <0.05) but not on waist–hip ratio, physical activity, or fruit and vegetable consumption. The participatory process appeared to be an effective means for stimulating change. The intervention may have slowed and perhaps reversed the tendency of adults to gain weight progressively with age.
Van Wier et al., 2009 [51]	N = 1386 workers from two IT-companies, two hospitals, an insurance company, a bank and a police force IG phone: *n* = 462 M/F = 321/141 Age Mean ± SD = 43 ± 8.8 BMI ± SD = 29.5 ± 3.5 IG internet: *n* = 464 M/F = 302/162 Age Mean ± SD = 43 ± 8.4 BMI ± SD = 29.6 ± 3.4 CG: *n* = 460 M/F = 306/154 Age Mean ± SD = 43 ± 8.7 BMI ± SD = 29.6 ± 3.7	Overweight	Netherlands	6 months	All groups received self-help materials (dealt with overweight, healthy diet and physical activity plus pedometer). Additionally, the IG received a lifestyle intervention program (10 modules about nutrition, physical activity…). IG phone: Received the program in a binder. Counseling by phone every two weeks. IG internet: Had access to an interactive website. Counseling by email when the employee finished a module. CG: Received only the self-help materials and no counseling.	Both groups had a significant decrease in weight loss (*p* < 0.001) and WC (*p* < 0.05 internet, *p* < 0.001 phone) in comparison with the CG. The difference between the intervention groups was not statistically significant. Weight loss intervention plus lifestyle counseling by phone and e-mail is effective for reducing body weight and WC. Furthermore, counseling by phone is effective for reducing fat intake and increasing physical activity.
Leslie et al., 2002 [52]	N = 122 petrochemical work-site (staff) IG1 Energy deficit diet (ED): *n* = 61 M/F: 61/0 Age Mean ± SD = 41.3 ± 8.1 BMI ± SD = 31.5 ± 3.7 IG2 Generalized low calorie diet (GLC): *n* = 61 M/F: 61/0 Age Mean ± SD = 42.1 ± 7.8 BMI ± SD = 30.4 ± 3.7	Overweight and obesity	UK	12 weeks weight loss plus 12 weeks weight maintenance	IG1 (ED): Individualized energy prescriptions (600 kcal subtracted from estimated daily energy requirements). Diet with and without meat. IG2 (GLC): They were given a 1500 kcal eating plan. Diet with and without meat. CG: Volunteers were randomized to different combinations (ED meat, ED no meat, GLC meat, GLC no meat). One-third of subjects were randomized to an initial control period prior to receiving dietary advice.	Both the ED and GLC groups had a significant mean weight loss at week 12 (*p* < 0.0001) in contrast with CG. But no difference was evident between diet groups in mean weight loss at 12 weeks (*p* = 0.34). The inclusion of lean red meat in the diet did not impair weight loss. The weight maintenance intervention was not effective, with a significant mean weight gain in all groups (*p* ≤ 0.003).
Pritchard et al., 1997 [53]	N = 58 business corporation employees IG (weight loss-diet-low fat): *n* = 18 Age Mean ± SD = 43.6 ± 6.0 M/F: 18/0 BMI ± SD = 29.0 ± 2.8 IG (weight loss-exercise): *n* = 21 Age Mean ± SD = 44.9 ± 6.5 M/F: 21/0 BMI ± SD = 29.2 ± 2.8 CG (weight maintenance): *n* = 19 Age Mean ± SD = 42.3 ± 4.5 M/F: 19/0 BMI ± SD = 28.6 ± 2.8	Overweight	Australia	12 months	IG (diet): Low fat intake (22% to 25% of energy) diet plus personalized dietary plan (on the basis of usual dietary pattern). IG (exercise): Subjects selected their own aerobic exercise regimen (realized in leisure time); minimum participation (three sessions of 30 min per week). CG: Monthly weight-monitoring sessions plus measurement protocol similar to those of the intervention groups and followed their usual pattern of activity and diet.	At 12 months the diet group was significantly different from baseline (*p* < 0.001) and from the CG in weight lost, BMI, total energy and total fat mass (*p* < 0.05). The dieters had greater weight loss than the exercise group (unsupervised aerobic exercise) (*p* < 0.05), as well as a lower BMI index.
Baer 1993 [54]	N = 70 management-level male employees IG: *n* = 33 M/F = 33/0 Age Mean ± SE = 44 ± 4.0 BMI ± SD = Not provided CG: *n* = 37 M/F = 37/0 Age Mean ± SE = 35 ± 3.0 BMI ± SD = Not provided	Coronary heart disease	USA	1 year	All subjects met with a registered dietitian who explained the results of the lipid analysis and discussed risk factors for coronary heart disease with an emphasis on diet. IG: Nutrition intervention: individualized instruction about the step 1 diet; group sessions (1 h every 3 months) on eating out, dietary fiber, and maintaining heart-healthy behaviors, and individualized follow-up by telephone (one call per month).	Significant decreases (*p* < 0.05) in total cholesterol, triglycerides, body weight and body fat were observed in intervention subjects at the 1-year follow-up. Although weight reduction was not a goal of the program, by decreasing energy intake and increasing energy expenditure, subjects lost weight and decreased body fat. The worksite provides many opportunities for dietetics professionals to conduct nutrition education programs to decrease risk factors associated with coronary heart disease.
Follick et al., 1984 [55]	N = 48 employees of a general hospital M/F: 41/7 Age range: 20–69 IG: *n* = 24 BMI ± SD = Not provided CG: *n* = 24 BMI ± SD = Not provided	Overweight	USA	18 weeks	IG: Weight loss program (14 session behavior modification program) plus incentive procedure. (5$ (×14) deposit was returned (one for each treatment session). CG: Weight loss program alone.	Both groups lost weight over the course of the intervention (*p* < 0.001) and there were no significant differences in weight loss between groups. The inclusion of an incentive procedure may improve the effectiveness of a behavioral weight loss intervention by decreasing attrition (*p* < 0.01).

BMI: Body mass index; CG: Control group; DI: Dietary intervention; ED: Energy deficit diet; EDM: Environmental dietary modification; GLC: Generalized low-calorie diet; IG: Intervention group; IP: Incentivized participants; lbs: pound-weight; LMW: Livin’ my weigh; M/F = Male/Female; MS: Metabolic syndrome; NE: Nutrition education; NIP: Non incentivized participants; NP: Not provided; PA: Physical activity; SD: Standard deviation; SE: Standard error; TMC: Telemedicine coaching; WC: Waist circumference; WM; Weight management.

**Table 2:** Assessment of study quality according to the 25-item CONSORT guidelines.

Author	1	2	3	4	5	6	7	8	9	10	11	12	13	14	15	16	17	18	19	20	21	22	23	24	25	Total	%
Thorndike et al. [22]	1	1	0.5	1	1	0.5	0.5	1	0	0	0.5	1	0.5	0.5	1	1	0.5	1	0	1	0	1	1	0	0	15.5	62
Röhling et al. [23]	1	1	0	1	1	0.5	0.5	1	1	0	0.5	1	1	0.5	1	1	0.5	1	1	1	1	1	1	1	1	20.5	82
Iturriaga et al. [24]	1	1	0.5	0.5	0	0.5	0	0.5	0	0	0.5	0.5	1	0.5	0	1	0.5	0	0	0	0	1	1	1	1	12	48
Day et al. [25]	1	1	0.5	0.5	0	0.5	0.5	0	0	0	0	0	1	0.5	1	1	0.5	0	0	1	0	1	1	0	0	11.5	46
Kempf et al. [26]	1	1	0	0.5	0	0.5	0.5	1	1	0	0.5	0.5	1	0.5	1	1	0.5	0	1	1	1	1	1	0	1	16.5	66
Tene et al. [27]	1	1	0.5	0.5	1	0.5	0	1	0	0	0.5	1	0	0.5	1	0	0.5	0	0	1	0	1	1	0	1	13	52
Viester et al. [28]	1	1	0.5	0.5	1	0.5	0.5	0.5	0	1	0.5	0.5	1	0	1	0	0.5	0	0	1	0	1	1	0	0	13	52
Shrivastava et al. [29]	1	1	0.5	0.5	1	0.5	0	0.5	0	0	0	0.5	1	0.5	1	1	0.5	0	0	1	0	1	1	0	1	14	56
Gepner et al. [30]	1	1	0.5	0.5	1	0.5	0.5	1	0	0	0.5	0.5	1	0.5	1	1	0.5	0	0	1	0	1	1	0	1	15	60
Faghri et al. [31]	0.5	1	0.5	0.5	1	0.5	0	0	0	0	0	0.5	0	0	1	0	0.5	0	0	1	0	1	0	0	1	9	36
Geaney et al. [32]	1	1	0.5	0.5	0	0.5	0.5	0.5	0	1	0	0.5	1	0	1	1	0.5	0	0	1	1	1	1	1	1	15.5	62
Solenhill et al. [33]	1	1	0	0	1	0.5	0	0	0	0	0	0	1	0	1	1	0.5	0	0	1	0	1	0	0	1	10	40
Mitchell et al. [34]	1	1	0.5	0.5	1	0.5	0.5	0.5	0	0	0.5	0.5	1	0	1	1	0.5	0	0	1	1	1	1	0	1	15.5	62
Fernández et al. [35]	1	1	0.5	0.5	1	0.5	0	0	0	0	0.5	0.5	1	0.5	1	1	0.5	0	0	1	1	1	0	0	1	13.5	64
Almeida et al. [36]	1	1	1	1	0	0.5	0	1	0	0	0	0.5	1	0.5	1	1	0.5	0	0	1	1	1	1	1	1	16	64
Østbye et al. [37]	0.5	1	1	1	1	0.5	0	0.5	0	0	0	0.5	0.5	0.5	1	1	0.5	0	0	1	0	1	1	0	1	14	56
Van Berkel et al. [38]	0.5	1	0.5	0.5	1	0.5	0.5	0	0	0	0	0.5	1	0.5	1	1	0.5	0	0	1	0	1	1	0	1	13	52
Mishra et al. [39]	1	1	0.5	1	1	0.5	0.5	0.5	0	0	0	0.5	1	0	1	1	0.5	0	0	1	0	1	0	0	0	12	48
Salinardi et al. [40]	0.5	1	0	0	0	0.5	0	0.5	0	0	0	1	1	0.5	1	1	0.5	1	0	0	0	1	0	0	0	9.5	38
Christensen et al. [41]	1	1	0.5	0.5	1	0.5	0	0.5	1	1	0.5	0.5	1	0.5	0	1	0.5	0	0	1	0	1	1	0	1	15	60
Thorndike et al. [42]	1	1	0	1	0	0.5	0.5	0.5	0	0	0	1	1	0	1	1	0.5	0	0	1	0	1	1	0	1	10	40
Linde et al. [43]	1	1	0.5	0.5	1	0.5	0	1	0	1	0	0.5	1	0	1	1	0.5	0	0	1	0	1	1	0	1	14	56
Nanri et al. [44]	1	1	0.5	0.5	0	0.5	0	0	0	0	0	0.5	1	0	1	1	0.5	0	0	1	1	1	1	0	1	12.5	50
Brehm et al. [45]	1	1	0.5	0.5	1	0.5	0	0.5	0	0	0	0.5	1	0	0	1	0.5	0	0	0	0	1	0	0	0	9	36
Christensen et al. [46]	1	1	0.5	0.5	1	0.5	0.5	0.5	1	1	0.5	0.5	1	0	1	1	0.5	0	0	0	1	1	1	0	0	12	48
Barham et al. [47]	0.5	1	0	0	1	0.5	0	0	0	0	0	0.5	0.5	0	1	0	0.5	0	0	0	0	1	0	0	0	6.5	26
Ferdowsian et al. [48]	0.5	0.5	0	0.5	0	0	0	0	0	0	0	0.5	1	0	0	1	0.5	0	1	1	0	0	1	0	1	8.5	34
Maruyama et al. [49]	1	1	0.5	0	0	0.5	0	0.5	0	1	0	0.5	1	0.5	1	1	0.5	0	0	1	0	1	0	0	1	13	52
Siegel et al. [50]	1	1	0	0	0	0.5	0	0	0	0	0	0	0.5	0	1	1	0.5	0	0	0	1	1	0	0	1	9.5	38
Van Wier et al. [51]	1	1	1	0.5	0.5	0.5	0.5	0.5	0	0	0	0.5	1	0	1	1	0.5	0	0	1	0	1	1	0	1	15	56
Leslie et al. [52]	0.5	1	0.5	0.5	0.5	0.5	0.5	0	1	0	0	0.5	0.5	0	1	0	0.5	0	0	0	0	1	0	0	1	9	36
Pritchard et al. [53]	0.5	1	0	0	0	0.5	0	0	0	0	0	1	1	0	1	1	0.5	0	0	0	0	1	0	0	1	8.5	34
Baer [54]	1	0.5	0	0	0	0.5	0	0	0	0	0	0.5	0	0	1	0	0.5	0	0	0	0	1	0	0	0	6	24
Follick et al. [55]	0.5	1	0	0	0	0.5	0	0	0	0	0	0	0	0	0	0	0.5	0	0	0	0	1	0	0	0	3.5	14

**Table 3:** Summary leave-one-out, Baseline of Body Mass Index and interventions.

ID	Autor	Change Effect	Baseline BMI	Int	ID	Autor	Change Effect	Baseline BMI	Int
1	Röhling et al.	0.53	3	1	19	Solenhill et al.	0.57	2	3
2	Christensen et al.	0.54	3	1	20	Solenhill et al.	0.58	2	3
3	Leslie et al.	0.53	3	1	21	Mitchell et al.	0.55	2	3
4	Leslie et al.	0.53	3	1	22	Mitchell et al.	0.53	2	3
5	Pritchard et al.	0.52	2	1	23	Almeida et al.	0.58	3	3
6	Pritchard et al.	0.54	2	1	24	Almeida et al.	0.58	3	3
7	Thorndike et al.	0.58	2	2	25	Østbye et al.	0.56	3	3
8	Thorndike et al.	0.58	2	2	26	Østbye et al.	0.56	3	3
9	Geaney et al.	0.57	2	2	27	Salinardi et al.	0.46	3	3
10	Geaney et al.	0.57	2	2	28	Christensen et al.	0.51	3	3
11	Geaney et al.	0.56	2	2	29	Thorndike et al.	0.55	2	3
12	Fernández et al.	0.55	2	2	30	Thorndike et al.	0.56	2	3
13	Brehm et al.	0.59	2	2	31	Nanri et al.	0.55	2	3
14	Brehm et al.	0.59	2	2	32	Maruyama et al.	0.55	2	3
15	Brehm et al.	0.59	2	2	33	Siegel et al.	0.59	2	4
16	Viester et al.	0.58	2	3	34	Mishra et al.	0.52	3	5
17	Viester et al.	0.58	2	3	35	Iturriaga et al.	0.56	1	5
18	Shrivastava et al.	0.55	2	1	Pooled		0.55		

**Table 4:** Moderator analysis, adjusted meta-regression by the baseline of Body Mass Index and interventions.

Variable	Baseline BMI	Sig.	Int-1	Sig.	Int-2	Sig.	Int-3	Sig.	Int-4	Sig.	Int-5	Sig.
INTERCEPT	1.36	<0.01	−0.39	<0.01	−0.81	<0.01	−0.52	<0.01	−0.59	<0.01	−0.52	<0.01
Coef	−0.85	<0.01	−1.26	<0.01	0.94	<0.01	−0.11	0.37	0.55	0.12	−0.44	0.03
Adjusted	Baseline BMI	Sig.	Int-1	Sig.	Int-2	Sig.	Int-3	Sig.	Int-4	Sig.	Int-5	Sig.
INTERCEPT	0.47	0.16										
Coef	−0.48	<0.01	−0.87	<0.01	0.62	<0.01	0.13	0.59	0.48	0.21	−0.46	0.08
